# Interim Recommendations from the Advisory Committee on Immunization Practices for the Use of Bivalent Booster Doses of COVID-19 Vaccines — United States, October 2022

**DOI:** 10.15585/mmwr.mm7145a2

**Published:** 2022-11-11

**Authors:** Hannah G. Rosenblum, Megan Wallace, Monica Godfrey, Lauren E. Roper, Elisha Hall, Katherine E. Fleming-Dutra, Ruth Link-Gelles, Tamara Pilishvili, Jennifer Williams, Danielle L. Moulia, Oliver Brooks, H. Keipp Talbot, Grace M. Lee, Beth P. Bell, Matthew F. Daley, Sarah Meyer, Sara E. Oliver, Evelyn Twentyman

**Affiliations:** ^1^National Center for Immunization and Respiratory Diseases, CDC; ^2^Watts Healthcare Corporation, Los Angeles, California; ^3^Vanderbilt University School of Medicine, Nashville, Tennessee; ^4^Stanford University School of Medicine, Stanford, California; ^5^University of Washington, Seattle, Washington; ^6^Institute for Health Research, Kaiser Permanente Colorado, Denver, Colorado.

Four COVID-19 vaccines are currently approved for primary series vaccination in the United States under a Biologics License Application or authorized under an emergency use authorization (EUA) by the Food and Drug Administration (FDA), and recommended for primary series vaccination by the Advisory Committee on Immunization Practices (ACIP): 1) the 2- or 3-dose monovalent mRNA BNT162b2 (Pfizer-BioNTech, Comirnaty) COVID-19 vaccine; 2) the 2- or 3-dose monovalent mRNA mRNA-1273 (Moderna, Spikevax) COVID-19 vaccine; 3) the single-dose adenovirus vector-based Ad26.COV.S (Janssen [Johnson & Johnson]) COVID-19 vaccine; and 4) the 2-dose adjuvanted, protein subunit–based NVX-CoV2373 (Novavax) COVID-19 vaccine. The number of doses recommended is based on recipient age and immunocompromise status (*1*). For additional protection, FDA has amended EUAs to allow for COVID-19 booster doses in eligible persons (*1*). Because COVID-19 vaccines have demonstrated decreased effectiveness during the period when the Omicron variant (B.1.1.529) of SARS-CoV-2 predominated, bivalent booster doses (i.e., vaccine with equal components from the ancestral and Omicron strains) were considered for the express purpose of improving protection conferred by COVID-19 vaccine booster doses (*2*). During September–October 2022, FDA authorized bivalent mRNA vaccines for use as a booster dose in persons aged ≥5 years who completed any FDA-approved or FDA-authorized primary series and removed EUAs for monovalent COVID-19 booster doses (*1*). Pfizer-BioNTech and Moderna bivalent booster vaccines each contain equal amounts of spike mRNA from the ancestral and Omicron BA.4/BA.5 strains. After the EUA amendments, ACIP and CDC recommended that all persons aged ≥5 years receive 1 bivalent mRNA booster dose ≥2 months after completion of any FDA-approved or FDA-authorized monovalent primary series or monovalent booster doses.*

Since June 2020, ACIP has convened 33 public meetings to review data relevant to the potential use of COVID-19 vaccines.^†^ The ACIP COVID-19 Vaccine Work Group (Work Group), comprising experts in adult and pediatric medicine, infectious diseases, vaccinology, vaccine safety, public health, and ethics, has met weekly to review COVID-19 surveillance data, evidence for vaccine efficacy, postauthorization effectiveness, safety, and implementation considerations for COVID-19 vaccines. To assess the certainty of evidence for benefits and harms of a bivalent booster dose and guide deliberations, ACIP used the Evidence to Recommendations (EtR) Framework.^§^ Within this framework, ACIP considered the importance of COVID-19 as a public health problem, including during the Omicron-predominant period, and issues of resource use, benefits and harms, patients’ values and preferences, acceptability, feasibility, and equity for use of the vaccines.

Effectiveness of monovalent COVID-19 vaccines was high after vaccine introduction in late 2020. However, declines in vaccine effectiveness (VE) against infection and COVID-19–associated hospitalization have been observed because of waning protection over time and differences between the virus for which the initial vaccines were designed and currently circulating variants. The Omicron variant, which emerged in November 2021, has increased immune evasion compared with that of earlier variants (*2*). During the Omicron-predominant period, monovalent mRNA primary series VE against SARS-CoV-2 infection and COVID-19–associated hospitalization was substantially lower and waned over time since vaccination (*3*). A third monovalent (booster) dose provided increased protection against infection and severe disease during the period of Omicron predominance, but VE of monovalent booster doses against COVID-19–associated hospitalization has also waned over time since receipt of the booster dose, especially during the recent BA.2/BA.2.12.1 and BA.4/BA.5 sublineage–predominant periods (*3*,*4*).

The goal of a bivalent booster vaccination is to expand the immune response to the currently circulating Omicron variant^¶^ and improve protection conferred by COVID-19 vaccines against severe disease (*2*,*5*). Specifically, the bivalent booster vaccines authorized by FDA contain mRNA encoding the viral spike (S) glycoprotein of SARS-CoV-2 Wuhan-hu-1 strain (ancestral) and the identical S glycoprotein of SARS-CoV-2 Omicron variant BA.4 and BA.5 (Omicron BA.4/BA.5) sublineages (*1*).

At the September 1, 2022, ACIP meeting, committee members reviewed evidence demonstrating monovalent VE against COVID-19–associated hospitalization during the BA.4/BA.5 period among immunocompetent adults aged ≥18 years of 49% (95% CI = 20%–68%) at 14–149 days after dose 3 and 34% (95% CI = 25%–42%) ≥150 days after dose 3 (*5*). Among evidence reviewed for children aged 5–11 years, monovalent VE against emergency department and urgent care visits for COVID-19 was 51% (95% CI = 34%–64%) at 14–59 days after dose 2 and declined to 18% (95% CI = −4% to 35%) ≥150 days after dose 2; among adolescents aged 12–17 years, VE against emergency department and urgent care visits was 63% (95% CI = 48%–73%) ≥7 days after dose 3. These data indicate that VE has declined during a time when the vaccine and circulating variants are different from the version of the virus against which the vaccines were designed to protect; bivalent booster doses might have the potential to improve vaccine protection against newly circulating Omicron variants.

ACIP recommendations for a COVID-19 bivalent mRNA booster dose were also guided by data on immunogenicity and safety from clinical trials of the Moderna and Pfizer-BioNTech bivalent vaccines composed of ancestral and Omicron BA.1 strains (*5*). The Moderna and Pfizer-BioNTech clinical trials included 437 and 315 participants who received 50 *μ*g Omicron BA.1–containing bivalent boosters and 30 *μ*g Omicron BA.1–containing bivalent boosters, respectively.** Among adults aged ≥18 years, geometric mean ratios (GMRs) of neutralization titers 28 days after Moderna bivalent (ancestral and BA.1 variant) booster dose were 1.2-fold higher for ancestral SARS-CoV-2 antibodies and 1.8-fold higher for Omicron SARS-CoV-2 antibodies compared with titers in those receiving a Moderna monovalent booster dose, thereby meeting superiority criteria.^††^ Among adults aged >55 years, GMRs of neutralization titers 1 month after a Pfizer-BioNTech bivalent (ancestral and BA.1 variant) booster dose were equivalent for ancestral SARS-CoV-2 antibodies and 1.6-fold higher for Omicron SARS-CoV-2 antibodies compared with titers in persons receiving a Pfizer-BioNTech monovalent booster dose, meeting noninferiority criteria^§§^ against the ancestral strain and superiority criteria against Omicron.

In the clinical trials of the Moderna and Pfizer-BioNTech bivalent (ancestral and BA.1 variant) booster doses, rates of local or systemic adverse events occurred with similar or lower frequency after a bivalent booster dose than after the second dose of a primary series with the same vaccine (i.e., homologous monovalent booster dose). No serious adverse events related to the vaccine were reported for mRNA COVID-19 updated bivalent booster doses (*5*). A rare risk for myocarditis and pericarditis has been identified after mRNA COVID-19 vaccination, primarily in adolescent and young adult males (*5*). The risk after a bivalent booster dose is not known; however, the observed risk for myocarditis and pericarditis after monovalent mRNA COVID-19 booster doses is similar to or lower than the risk after dose 2 of the primary series. Regular review of safety data, including myocarditis and pericarditis risk after bivalent booster doses, will continue in national safety surveillance systems. Modeling scenarios reviewed during the ACIP meeting showed that, irrespective of the presence of a new variant, vaccination coverage in adults aged ≥18 years similar to coverage for influenza vaccine would lead to a reduction in hospitalizations and deaths of >20% and >15%, respectively, compared with a recommendation for adults aged ≥50 years only (*3*). In addition, absent a new variant, booster doses administered to adults aged ≥18 years in September 2022 were projected to prevent 137,000 more hospitalizations and 9,700 more deaths compared with those prevented by booster doses administered in November 2022.^¶¶^

Data to guide the pediatric expansions (i.e., to include children aged ≥5 years) for bivalent mRNA COVID-19 vaccines included data on monovalent boosters in both the pediatric and adolescent populations and data on bivalent boosters in the adult population. Recommendations for monovalent booster doses of Pfizer-BioNTech were discussed at previous ACIP meetings and were based on 1) safety and immunogenicity of the booster dose, 2) postauthorization safety data after a primary series in children and adolescents and booster doses in adults, and 3) waning VE after a primary series during the Omicron-predominant period.*** Safety and immunogenicity data for monovalent Moderna COVID-19 booster dose vaccination in children aged 6–11 years and adolescents aged 12–17 years were also reviewed by CDC and FDA. Antibody levels obtained 28 days after a Moderna monovalent booster dose compared with titers 28 days after receiving a Moderna primary series in young adults aged 18–25 years demonstrated neutralization titers 4.2-fold and 5.1-fold higher in children aged 6–11 years and adolescents aged 12–17 years, respectively (*6*). Reactogenicity symptoms were similar to those observed after receipt of booster doses in adults (*6*).

ACIP also examined data pertaining to equity in consideration of each EtR domain, in line with the COVID-19 ACIP Work Group's approach to the EtR Framework through the lens of health equity (*3*). Data reviewed pertaining to health equity included 1) the disproportionate incidence of COVID-19 illness, hospitalization, and death among persons of racial and ethnic minority groups; 2) the demographic characteristics of clinical trial populations compared with those of the U.S. population; and 3) the evidence of persistent inequity in receipt of primary series and booster doses, with potential drivers including differences in access, differences in acceptability, and evidence of limitations to feasibility of booster dose implementation.

In its deliberations at the September 1, 2022, meeting, ACIP discussed the rationale for a bivalent booster vaccine for persons in all age groups previously recommended to receive monovalent booster doses. ACIP concluded that the evidence reviewed, including data and considerations from the EtR Framework, supported use of a dose of a bivalent booster dose of mRNA COVID-19 vaccine in eligible recipients of a COVID-19 primary vaccination series (Box). Since FDA removed the EUAs for mRNA monovalent COVID-19 vaccine booster doses, ACIP repealed its previous recommendations for administration of monovalent Pfizer-BioNTech and Moderna monovalent boosters for persons aged ≥12 years and adults aged ≥18 years, respectively. During this discussion, ACIP voting members and liaisons underscored the importance of extending the potential benefits of the Omicron BA.4/BA.5–targeting bivalent vaccines to pediatric populations. On October 12, 2022, FDA authorized and CDC recommended bivalent Pfizer-BioNTech booster doses for children aged 5–11 years. After the EUA amendments, ACIP and CDC recommended that all persons aged ≥5 years should receive 1 bivalent mRNA booster dose ≥2 months after completion of any FDA-approved or FDA-authorized monovalent primary series or monovalent booster dose (Table). The pediatric data described above were reviewed again at the ACIP meeting on October 19, 2022. In addition, persons who recently had a SARS-CoV-2 infection may consider delaying a primary series dose or booster dose by 3 months from symptom onset or a positive test result (if infected person was asymptomatic). Additional supporting evidence for the EtR Framework is available at https://www.cdc.gov/vaccines/acip/recs/grade/covid-19-bivalent-booster-etr.html and complete interim clinical considerations are available at https://www.cdc.gov/vaccines/covid-19/clinical-considerations/covid-19-vaccines-us.html.

BOX **Timeline of COVID-19 bivalent vaccine authorizations by Food and Drug Administration and CDC vaccination recommendations — United States, fall 2022**

**Authorizations and vaccine recommendations**

**August 2022**

- FDA authorizes Pfizer-BioNTech and Moderna bivalent (ancestral and Omicron BA.4/BA.5 variant) COVID-19 vaccines for use as booster doses.

**September 2022**

- CDC recommends Pfizer-BioNTech and Moderna bivalent booster vaccines for persons aged ≥12 years (Pfizer-BioNTech) and adults aged ≥18 years (Moderna) ≥2 months after last primary series or booster dose.

**October 2022**

- FDA revises EUA to authorize a Pfizer-BioNTech bivalent booster dose for persons aged ≥5 years; CDC recommends bivalent boosters in children aged 5–11 years ≥2 months after last primary series or booster dose.
- FDA authorizes and CDC recommends a Moderna bivalent booster dose for children and adolescents aged 6–17 years ≥2 months after last primary series or booster dose.
- FDA authorizes and CDC recommends a monovalent Novavax booster dose for adults aged ≥18 years instead of a bivalent booster if they have completed the primary series vaccination but have not previously received a COVID-19 booster, and if they cannot or will not receive mRNA vaccines.

**Abbreviations:** EUA = emergency use authorization; FDA = Food and Drug Administration.

**TABLE:** COVID-19 vaccines approved or authorized for emergency use by the Food and Drug Administration and recommended by the Advisory Committee on Immunization Practices for persons aged ≥6 months — United States, October 2022*

Age	Primary series (timing of vaccination)		Bivalent booster dose^†^
	For most persons	For moderately or severely immunocompromised persons	
**6 mos–4 yrs**	2-dose Moderna (0, 4–8 wks) or	3-dose Moderna (0, 4, 8 wks) or	No booster dose authorized
	3-dose Pfizer-BioNTech (0, 3–8, 11–16 wks)	3-dose Pfizer-BioNTech (0, 3, 11 wks)	
**5 yrs**	2-dose Moderna (0, 4–8 wks) or	3-dose Moderna (0, 4, 8 wks) or	Pfizer-BioNTech
	2-dose Pfizer-BioNTech (0, 3–8 wks)	3-dose Pfizer-BioNTech (0, 3, 7 wks)	
**6–11 yrs**	2-dose Moderna (0, 4–8 wks) or	3-dose Moderna (0, 4, 8 wks) or	Moderna or Pfizer-BioNTech
	2-dose Pfizer-BioNTech (0, 3–8 wks)	3-dose Pfizer-BioNTech (0, 3, 7 wks)	
**12–17 yrs**	2-dose Moderna (0, 4–8 wks) or	3-dose Moderna (0, 4, 8 wks) or	Moderna or Pfizer-BioNTech
	2-dose Novavax (0, 3–8 wks) or	2-dose Novavax (0, 3 wks) or	
	2-dose Pfizer-BioNTech (0, 3–8 wks)	3-dose Pfizer-BioNTech (0, 3, 7 wks)	
***≥*18 yrs^§^**	2-dose Moderna (0, 4–8 wks) or	3-dose Moderna (0, 4, 8 wks) or	Moderna or Pfizer-BioNTech Novavax monovalent booster may be used in limited situations^¶^
	2-dose Novavax (0, 3–8 wks) or	2-dose Novavax (0, 3 wks) or	
	2-dose Pfizer-BioNTech (0, 3–8 wks)	3-dose Pfizer-BioNTech (0, 3, 7 wks)	

**Abbreviation:** FDA = Food and Drug Administration.

* https://www.cdc.gov/vaccines/covid-19/clinical-considerations/interim-considerations-us.html

^† ^Bivalent booster doses are authorized ≥2 months after the last primary series or monovalent booster dose. The Novavax monovalent booster is authorized ≥6 months after the last primary series dose.

^§ ^For primary series vaccination, Pfizer-BioNTech, Moderna, and Novavax COVID-19 vaccines are recommended. Janssen (Johnson & Johnson) should only be used in very limited situations because of the risk for thrombosis with thrombocytopenia syndrome after receipt of the vaccine. https://www.cdc.gov/mmwr/volumes/71/wr/mm7103a4.htm

^¶ ^A monovalent Novavax booster dose (rather than a bivalent mRNA booster dose) may be used in limited situations in adults aged *≥*18 years who completed any FDA-approved or FDA-authorized monovalent primary series, have not received any primary booster doses, and are unable to receive an mRNA vaccine (i.e., mRNA vaccine is contraindicated or not available) or are unwilling to receive an mRNA vaccine and would otherwise not receive a booster dose.

ACIP emphasized that achieving high and equitable coverage with a COVID-19 primary vaccination series remains the highest priority and is fundamental to reducing COVID-19–related morbidity and mortality, including in younger age groups with lower vaccination coverage. ACIP also stressed the importance of ensuring global equity in access to COVID-19 vaccines for the prevention of disease in vulnerable persons and mitigation of the emergence of SARS-CoV-2 variants.

After authorization by FDA on October 19, 2022, CDC recommended use of a monovalent Novavax booster dose (rather than a bivalent mRNA booster dose) in limited situations. These situations include use in adults aged ≥18 years who completed any FDA-approved or FDA-authorized monovalent primary COVID-19 vaccination series, have not received any previous booster doses, and are unable to receive an mRNA vaccine (i.e., mRNA vaccine is contraindicated or not available) or unwilling to receive an mRNA vaccine and would otherwise not receive a booster dose (*7*). Additional supporting evidence for the EtR is available at https://www.cdc.gov/vaccines/acip/recs/grade/covid-19-novavax-adult-booster-etr.html.

Before vaccination, providers should provide the EUA Fact Sheet for the vaccine being administered and counsel vaccine recipients about expected systemic and local reactogenicity. Additional clinical education materials are available at https://www.cdc.gov/vaccines/covid-19/index.html, including additional clinical considerations at https://www.cdc.gov/vaccines/covid-19/clinical-considerations/covid-19-vaccines-us.html. These interim ACIP recommendations and clinical considerations are based on bivalent booster doses of COVID-19 vaccine and might change as more evidence becomes available. At the September meeting, existing recommendations for persons who are immunocompromised were highlighted, including preexposure prophylaxis with the medication Evusheld, a combination of two monoclonal antibodies (tixagevimab and cilgavimab) administered every 6 months to persons who are or become moderately or severely immunocompromised, to supplement vaccine-conferred protection (*5*). 

## Reporting of Vaccine Adverse Events

Adverse events occurring after receipt of any COVID-19 vaccine should be reported to the Vaccine Adverse Event Reporting System (VAERS) at https://vaers.hhs.gov or 1-800-822-7967. Vaccination providers are required under the provisions of the provider agreements for the CDC COVID-19 Vaccination Program and by FDA to report vaccine administration errors, serious adverse events, cases of multisystem inflammatory syndrome, cases of myocarditis, cases of pericarditis, and cases of COVID-19 that result in hospitalization or death after administration of COVID-19 vaccine under EUA. Health care providers are encouraged to report any clinically significant adverse event, even if it is unclear whether the vaccine caused the event. In addition, CDC has developed v-safe, a voluntary, smartphone-based active surveillance system that monitors adverse events occurring after COVID-19 vaccination. Reports to v-safe indicating a medically significant health impact are followed up by CDC’s v-safe call center to collect additional information and complete a VAERS report, if indicated. Information on v-safe is available at https://www.cdc.gov/vsafe.

**Summary**

**What is already known about this topic?**

In the United States, COVID-19 monovalent booster vaccination was previously recommended, but related protection decreased after the emergence of Omicron subvariants.

**What is added by this report?**

In fall 2022, CDC recommended a bivalent mRNA COVID-19 vaccine booster dose for persons aged ≥5 years, administered ≥2 months after completing the primary series or after receipt of a monovalent booster dose.

**What are the implications for public health practice?**

Bivalent COVID-19 vaccine booster doses might improve protection against SARS-CoV-2 Omicron sublineages and, along with completion of a primary series in persons who remain unvaccinated, are important to protect against COVID-19, particularly among those persons who are at increased risk for severe illness and death.

## Footnotes

* On September 1, 2022, ACIP voted in favor of the following interim recommendations: 1) 13 to one in favor of a single dose of bivalent Pfizer-BioNTech COVID-19 vaccine for persons aged ≥12 years ≥2 months after receipt of a primary series or prior monovalent booster dose, under the EUA issued by FDA. ACIP repealed its previous recommendations for administration of monovalent Pfizer-BioNTech COVID-19 vaccine boosters for persons aged ≥12 years; 2) 13 to one in favor of recommending a single dose of bivalent Moderna COVID-19 vaccine for adults aged ≥18 years ≥2 months after receipt of a primary series or prior monovalent booster dose, under the EUA issued by FDA. ACIP repealed its previous recommendations for administration of monovalent Moderna COVID-19 vaccine boosters for adults aged ≥18 years. On October 12, 2022, FDA authorized bivalent booster doses from Pfizer-BioNTech for children aged 5–11 years, and CDC recommended expanding the recommendation for receipt of a single dose of bivalent Pfizer-BioNTech COVID-19 vaccine for persons aged ≥5 years.

†
https://www.cdc.gov/vaccines/hcp/acip-recs/vacc-specific/covid-19.html

§
https://www.cdc.gov/vaccines/acip/recs/grade/downloads/acip-evidence-recs-framework.pdf

¶ https://covid.cdc.gov/covid-data-tracker/#variant-proportions (Accessed October 19, 2022).

** In the clinical trials, the Moderna booster contained 25 *μ*g ancestral mRNA spike protein and 25 *μ*g of Omicron BA.1 mRNA spike protein (total = 50 *μ*g) and Pfizer-BioNTech contained 15 *μ*g ancestral mRNA spike protein and 15 *μ*g Omicron BA.1 mRNA (total = 30 *μ*g).

†† Superiority is considered met when the lower bound of the 97.5% CI of the GMR, the ratio of neutralization titers in the intervention versus the control group, is >1.

§§ Noninferiority is considered met when the lower bound of the 97.5% CI of the GMR is ≥0.67.

¶¶ https://covid19scenariomodelinghub.org/viz.html (Accessed August 18, 2022).
